# Moving Beyond Clinical Imaginaries: Technogeographies of the Everyday Urban

**DOI:** 10.1007/s10912-024-09872-y

**Published:** 2024-07-13

**Authors:** Daryl Martin, Dara Ivanova, Thorben Peter Høj Simonsen

**Affiliations:** 1 University of York, York, UK; 2 Department of Architecture, TU Delft, Delft, The Netherlands; 3 The Danish Center for Social Science Research, Copenhagen, Denmark

**Keywords:** Care, Place, Health apps, Everyday urbanism, Care infrastructures

## Abstract

In this paper, we analyse the intersections between care and place in mundane spaces not explicitly designed for the provision of care, and where digital technologies are used to mediate ecologies of distress in the city. We locate our analysis alongside studies of how digital technologies impact the experience of care within non-clinical spaces, whilst noting that much research on the use of technologies for care remains haunted by clinical imaginaries. Bringing together ideas of multi-sited therapeutic assemblages, technogeographies of care, and how places-by-proxy can act as conduits for care, we explore an example of an online app being used in public space to manage experiences of anxiety in an everyday urban environment. We reflect on this illustrative example to trace the movement of care as it is mediated through digital technologies—out of the clinic, beyond the home, and into the ordinary spaces of the city. We conclude that the entanglements of digital technologies and ordinary urban places prompt us to entirely reconsider questions of the *where* of care.

## Introduction

In this paper, we develop an understanding of the dynamic intersections between care and place, especially in mundane spaces not explicitly designed for the provision of care, but where digital technologies are increasingly used to mediate ecologies of distress (e.g. Goodings and Tucker 2018). As we will argue, it is in the ordinary spaces of the city that a renewed analysis of the intersections of care and place should be focused (Ivanova 2020a). Indeed, mundane settings are effective entry points for research that seeks to understand the role of digital technologies in the lives of their users (Leszczynski 2020). In doing so, we situate our work within debates that disrupt the spatial imaginaries and temporal regimes that are typically associated with healthcare—the space–time of the clinic, perhaps—and instead concentrate on practices of care that happen outside the hospital or other formal institutional settings (Langstrup 2013; Langstrup et al. 2013). We locate our arguments alongside scholars of technologically mediated care who argue for a move from thinking in terms of digital networks that are focused on abstract notions of the patient to geographically tracing the dispersed spatial practices of care (Oudshoorn 2011); by doing so, we can offer an analysis of how technologies fundamentally reshape our understandings of the mundane places in which they are used and the behaviours of those using them (Lehoux et al. 2004; Trnka 2021). In this, we are especially inspired by Oudshoorn’s notion of the technogeography of care, a phrase that captures how “technologies contribute to creating interdependencies and distributing responsibilities between people, places and technical devices, thus reconfiguring who cares,” as well as “connecting previously distinct places, redefining the meaning of these places, and creating new sites where care takes place” (2012, 124).

Previous research on the geographies of care has highlighted the role of domestic space in anchoring contemporary care practices (Milligan 2001) and, moreover, enlisting patients and their carers into new practices and responsibilities in the management of chronic conditions (Langstrup 2013). In her study of haemophilia and asthma patients, Langstrup found that domestic spaces were so integral to the routine management of their care that the home becomes, in effect, “an extension of the clinic” (2013: 1014), echoing arguments that the use of new medical technologies creates new forms of healthcare space within settings previously separate from clinical space (Schillmeier and Domènech 2010). For Langstrup and colleagues, care work is “highly collaborative” in character (2013, 51), especially with the use of online technologies that facilitate virtual encounters between patient and clinician. Spatially, the consequence of this shared work is that we should understand healthcare encounters as corporate rather than private in character, with the home “made available at times, by technology, patients, [and] spouses, as a place that can become reconfigured as a part of the corporate ecologies of care” (Langstrup et al. 2013, 56). The reconfiguration of domestic space by medical technologies clashes with aesthetic ideals of home (Angus et al. 2005); when home becomes the site of long-term and everyday care, the technologies that facilitate this shift are often experienced as disruptive and unwelcome (Weiner and Will 2018). This is because medical technologies are experienced as distinct from other home appliances; moreover, the telecare device “introduces a technogeographical configuration of care in which the home is transformed into a place where patients are made responsible for monitoring their own bodies” (Oudshoorn 2012, 129). These technologies enlist carers, both formal and informal, into extended systems of care, surveillance, and spatial ordering, albeit in unpredictable and often resistant ways (Oudshoorn 2012). The result is that healthcare technologies accelerate and intensify the ambiguous nature of non-clinical space, due to the incorporation of medical norms, rhythms, and practices.

We might make two broad observations about these studies of how digital technologies impact the experience of care within non-clinical spaces: namely, the absent presence of the clinic within these studies, and the overwhelming focus on how domestic settings have been re-shaped by clinical norms and medical devices. These two observations are related; the literature on telecare within health geography and science and technology studies is somewhat haunted by clinical imaginaries because of the (understandable) focus on the disruption to domestic practices in single sites in much of this literature. In her notion of the multi-sited therapeutic assemblage, Trnka has recently called for a re-evaluation of the idea of the therapeutic landscape (cf. Gesler 1992; Williams 2017), and how it plays out in a wide diversity of sites, so that the therapeutic “comes to be understood as neither anchored to a single site nor constituted through the inter-relation of home and clinic, but defined via a (shifting, dynamic, temporal) assemblage of multiple places, people, and resources” (Trnka 2021). It has long been argued by scholars of relational care that we need to rethink the spatial contexts of care, especially to think outside of domestic norms and practices (Tronto 2013); certainly, Trnka’s definition articulates a wider, and deeply ambivalent, milieu of care. One of the most fascinating examples in Oudshoorn’s research (2012) relates to how mobile electrocardiogram recorders acted as an “indiscreet technology” in public space, as the sounds they made to signal potential heart problems embarrassed their wearers and violated any sense of their privacy: as Oudshoorn noted, the alerts this telecare device emitted “was part of a technogeography of care that transformed shopping malls and trains into scary spaces in which patients’ failing bodies were exposed to others” (2012, 135). The breaches between public and private space illustrated by this example opens up a wider set of spatial questions for the role of the digital and the experience of place in contemporary practices of care. That is, when exploring how questions of place and care intersect and overlap, especially when these are related to the use of portable digital technologies, we need to move beyond clinical imaginaries to understand how these technologies work in public spaces that are, by their character, inherently plural, contingent, contentious, and layered places. Moreover, given the (self-)monitoring of health data via apps on commercially ubiquitous devices rather than exclusively medical technologies (Lupton 2017; 2019), we need to re-adjust our understandings of how care is managed, delivered, and experienced in very mundane ways (Pink et al. 2014). We need to take Puig de la Bellacasa’s advice for a disruptive understanding and relational understanding of care that comes with thinking “*in* the world” (2017, 10); within the context of contemporary cities, we need to understand the material, social, and affective changes to place because of the role of digital technologies situating questions of care within the urban experience, and how these may, or may not, enable processes of recovery and wellbeing (Duff 2011; 2012).

To this end, we will proceed with a short review of literature within health geography, medical sociology, and science and technology studies that has focused on questions of place and technology in relation to practices of care, before exploring a case study of the Minddistrict app to explore the dispersal of care-infrastructures within public space (see also Langstrup 2013). We use an illustrative example, based on reporting from the Danish broadcaster DRTV of how one young woman used this app to mediate her fear of using stairs and escalators in public space, as a way of analysing the use of mundane technologies within the built environment more generally. The increasing navigation of anxiety, illness, and public space via digital technologies, accessed through smartphones, prompts us to reevaluate established understandings of care, place, and ideas of care *happening within specific places*. In light of the ubiquity of digital technologies in how people move in, and through, contemporary cities, in this paper we consider the implications for our conceptualisation of what it means to be *in place*—especially when related to experiences and practices of care. That is, we evaluate the affordances of digital technologies to re-imagine places in more open ways: rather than being treated as a way of subjugating their users, and tethering them to the clinical norms that can still haunt these technologies (Trnka 2021), and rather than considering digital technologies as somehow holding the potential to erase questions of place and usher in placeless cultures of care (cf. Ivanova and Simonsen 2023), we instead understand digital technologies in an infrastructural sense as connecting places within wider networks of caring practices, making them *places-by-proxy*, or conduits of care (Ivanova et al. 2019; Ivanova 2020a). We therefore articulate an understanding of *post-place care* (Ivanova 2020b), where our experiences of care are deeply enmeshed with our experience of place, which is in turn connected to complex digital infrastructures, and entangled within deeply ambivalent political and economic logics. Consequently, applying digital technologies to mediate the broader ecologies of distress in the city may afford the situated enactment of a wider milieu of care, which is ambiguous in its effects, and this may prompt us to transcend ideas of fixed localities in our search for the infrastructures that support new practices of care.

## Place and technology within practices of care

### Architecture, place, and the materialities of care

The importance of place with respect to the organisation of social life has been spelled out by Gieryn, who argues that “place is not merely a setting or a backdrop, but an agentic player in the game – a force with detectable and independent effects on social life” (2000, 466). Gieryn distinguishes place—which he defines as integrating the specifics of geographic location, material form *and* the investment of social meaning by its users—from space—which he considers in a more abstract and geometrical sense, rather like a place emptied of cultural meaning and value. Place, in this understanding, is always more than a specific geographical location, but rather a kind of framework through which to understand the social processes and encounters that play out within that location (Pink 2012). This leads to a dynamic understanding of place, rather than any sense of a passive landscape, with pre-existing qualities that are merely encountered and absorbed by its users (Duff 2011). It leads to a relational understanding that considers practices of care as connected with an expanded sense of citizenship (Kontos et al. 2017), including relations between the non-human and the human; as Puig de la Bellcasa notes, although care “is a human trouble,” this cannot mean it is “a human-only matter” (2017, 2). The ongoing relations between human and non-human agents within any particular space leads to a sense of place that is open rather than bounded, and subject to change (Cresswell 2004). With respect to the experience of health and wellbeing within particular places, Kearns and colleagues argue that “health-related resources are generated in place through the exercise of complex forms of agency – including people ‘acting into’ and ‘receiving from’ place – and are not static or inherent qualities” (Kearns et al. 2014, 108). As researchers interested in the intersections between place and care, this leads us to pay attention to the relations between agents, artefacts, and their environments that prompt particular practices and atmospherics of care (Latimer and Munro 2009).

Care is an always situated practice, whereby individual actions by specific individuals are animated within much wider networks (Schillmeier 2017, 59). Following Puig de la Bellacasa (2017), we seek to understand the ambivalences, complexities, and fluidities of care through a sensitivity to the non-human in the *doing* of care, and the places in which care is experienced and enacted. This entails an attention to the quiet objects that are less noticed in accounts of the work of carers (Pink et al. 2014). As Maller has argued, there is a need for researchers to go beyond obvious healthcare technologies in their studies of care, and rather to focus on mundane environments and routine objects instead (2015). This has led to Buse and colleagues’ call for an awareness of the materialities of care, whereby they argue that material objects and environments “are shared between people as part of practices of care, they sometimes ‘stand in’ for caring relations, and may shape, enable or constrain practices of caring”; in this understanding, “mundane materialities act as a lens for (re)examining care practices in health and social care contexts” (2018, 245). In their heuristic of the materialities of care, they argue for a focus on the spatialities of care (2018: 246–248) as a way of tracing how the built environment can heighten an awareness of care practices in everyday ways (see also Brownlie and Spandler (2018) on the cultures of support played out in routine ways within unremarkable urban settings).

One of the strands of research that has followed in light of these calls for an attention to the materialities of care has focused on the built environment, and the agency of the non-human in processes of wellbeing and recovery (Martin et al. 2019). This research often also looks to earlier studies, such as those of Gieryn, who in his important paper *What Buildings Do* (2002) offers a blueprint for thinking about how buildings both enable and constrain the agency of their users, and how these tensions in use can be traced back to debates and disputes in their design stages, involving architects, engineers, clients, and stakeholders. These tensions are also captured by Simonsen and Duff (2020), who tease out the dynamic relations between the designed spatial order of an inpatient setting and the emergent spatial orderings of staff and patients therein, showing how the therapeutic effects of healing architecture will be more a function of spatial orderings than any simple material causation. With its focus on the role of the environment in processes of recovery and wellbeing, the work of Cameron Duff has also been significant in steering a sensitivity to the different qualities of place in brokering practices and experiences of care (2011; 2012; 2016). In Duff’s theory of enabling places, the therapeutic experiences of their users are not determined by environmental factors in any predictable sense, but rather enabled as “a function of the novel practices and social interactions afforded therein” (2012, 1394), and through a combination of the material resources, affective qualities and social practices fostered by that place. Martin and Roe have used Duff’s work in their analysis of Maggie’s Centres (2022), a cancer support service with drop-in centres nestled within the grounds of large hospital sites. Their analysis of Maggie’s Centres as spaces of hope for their users builds on earlier papers that stress the use of particular furnishings and spaces to stage a sense of comfort in the charity’s buildings (Martin 2021), the complex orchestration of staff work and spatial design to create a non-institutional feel to the buildings (Martin 2016), and the efforts of architects, staff and centre users to evoke atmospherics of care (Martin et al. 2019). Whilst these studies offer a sense of how spatial design can be related to social practices, and the enmeshment of non-human elements in the delivery of care that is humanistic at its core, it remains the case that these studies are of particular and singular buildings. These are studies of buildings that, in their quest to avoid the atmospherics of the hospital (Butterfield and Martin 2016), can be said to be haunted by similar clinical imaginaries to those identified in our review of studies of telecare technologies above (Angus et al. 2005; Langstrup 2013). For this reason, we are mindful of the need to explore work that engages with wider urban environments, and includes an understanding of the role of digital technologies in building a contemporary sense of place.

### “Dwelling” within digital places

In many ways, it is the ability to *sense* the places we habitually inhabit that is being disrupted and that, we pose, should be understood anew. The fast and relentless spread of digitalisation and digital technologies in almost all social domains, particularly in the West, has launched a process of displacement, shifting many of our everyday interactions from the material to the digital. This process does not represent a simple replacement of material with digitalised environments, but rather forces us to reconsider and reconceptualise what a (good/bad) place is, and what it means to be *in place*. How should we think about places, now that digital technologies are colonising those places, changing them in ways we cannot yet fully comprehend? The relationship between oneself and the environment has long been theorised as an interface between humans’ spaces and their very being. For Heidegger (1951), rootedness within a locale can be understood as *dwelling*—a way of being as one with one’s surroundings, building within it (cf. Sennett 2018), and *keeping* things in place (Latimer and Munro 2009). Ingold (2011) sees the environment as a constant flux of materials; the places we inhabit coming to life as we move through them—“through a world in perpetual formation.” These ideas are helpful in understanding how we care for place in mundane ways. Yet, we must also consider how they sit next to virtual or sensory reality technologies as they are becoming an increasingly important mode of entertainment, communication, and caring in our societies. Can we dwell in digital places or do we simply enter and exit them? What elements of the world do we form—in Ingold’s words—as we move through an amalgamation of digital and material environments? Considering such questions is necessary when sketching a full picture of contemporary sense of place and its future, particularly for care.

The healthcare field has been particularly willing and able to adjust to, and incorporate, digitalisation within their practices. Imaginaries of digitalisation as a solution to all ills plaguing the system (Ivanova and Simonsen, 2023) have pushed forward narratives of placelessness as a way to conquer distance and alleviate (staff) shortages. Such promissory discourses of care going digital (Pickersgill 2011, 2019; Janssen 2016) go hand-in-hand with an erasure of place, because placement and re-placement are often seen as glitches in supplying care to patients quickly and efficiently. The materiality of place and its very presence become a problem to be overcome with the help of flashy technologies that deliver made-to-order care environments (Ivanova 2020b). These developments are congruent with what has been termed a digital turn in geography (Ash et al. 2018; Kinsley 2014) and have been critically analysed by STS and sociology of care scholars, who have argued that—unlike the promissory narratives of placelessness—care is always firmly placed in materialities and practices, delicately configured within networks and settings (Bell 2018; Ivanova et al. 2016; Martin et al. 2015; Langstrup 2013; Pols 2012; Oudshoorn 2011; Milligan 2001; Schillmeier and Domenech 2010; Mort et al. 2009). However fruitful this critique has been in opening up the thinking on place and care, the digital turn is becoming so pervasive both in the hospital and in everyday places, that it is crucial to think beyond the dichotomy of digital and material, in an attempt to consider these as elements that *together* make up the world, just as we move through it (Ingold 2018).

One way to conceptualise care as rooted in an amalgamation of digital and material practices is through the notion of *post-place care* (Ivanova 2020b). This term describes an extension of place into further (digital, affective, troubling, sensory) carescapes, and it insists that we come to terms with care places as fractured, layered, and open. Post-place care invites us to engage with an unfolding care geography, where digital technologies play a crucial role in emplacing one in their environment (differently). Yet, far from digital alone, post-place carescapes (Ivanova et al. 2016) should be understood as negotiated with ICT, care materialities, digital assemblages, and affective, sensory, or immersive experiences. An emphasis on only one of these elements would miss the fuller picture of this negotiation coming to sit—well or indeed uncomfortably—together. To care in/for post-place is therefore to settle in complexity, and work to *find* the place of care, as opposed to assuming its (fixed) locality.

We propose that this is an important step in any analysis of place and care for two reasons. Firstly, because technologies purporting placeless care are increasingly becoming dominant within healthcare, thus displacing care practices towards digital and ICT-produced spaces. The second reason lies in our understanding that carescapes—or places of care as active agents in the care process—are often not fixed within a particular location, but rather through intersecting (care) infrastructures. Thinking about *care infrastructures* decentres care from locations strictly considered caring (hospitals, clinics), focusing attention instead on places outside of care (Buse et al. 2018). Danholt and Langstrup (2012) have argued that care practices may be located within infrastructures forming heterogeneous assemblages, showing how an individual is always intertwined with people and things in a care assemblage. Their analysis goes beyond the locus of the individual to illuminate the infrastructures that allow and support caring. These infrastructures are often overlooked in healthcare studies, because they are not traditionally considered *caring*. As Buse et al. (2018) point out, “designers, architects, and planners can orchestrate environments where care may take place with intended and unintended consequences” (253). Within post-place care, digital carescapes are often being imagined and produced for the healthcare field by ICT specialists and start-up tech companies, whose imaginaries of placelesssness may be at odds with material, real environments (Ivanova 2020b; Ivanova and Simonsen 2023).

In tracing care places outside of care, it may be helpful to think of various infrastructures that make up places as *place-by-proxy* (Ivanova et al. 2019; Ivanova 2020a). The idea behind place-by-proxy is that particular spaces can become—by virtue of their being there—places that are conduits for care. In their analysis of a Dutch baby-foundling room, Ivanova et al. (2019) describe how the room becomes a place by virtue of the infrastructures that are attached to it, animating its existence by actions happening elsewhere. The baby foundling room itself is empty and unused, almost a theatre background piece, yet the fact of it being there, becomes a political, moral and affective care-issue, making the room a care place, despite its inherent emptiness. This case illustrates the importance of proxy infrastructures, which are part and parcel of the care process in very specific ways. This line of thinking may be helpful in making sense of digital and urban environments of care as a decentred—and perhaps dispersed—proxy to care locales. For our purposes here, we understand caring as a fractured and open set of activities, animated within a variety of infrastructures, in line with Trnka’s (2021) analysis of therapeutic landscapes and building upon Oudshoorn’s (2011) notion of technogeography of care.

## Minddistrict: a *place-by-proxy* creating a wider milieu of care

In the following discussion, we seek to move beyond traditional clinical imaginaries, and the institutional spaces of care that may support and sustain such imaginaries, and instead aim to consider the role of digital technologies in brokering wider ecologies of distress in the city. As such, we now turn to the example of internet-based treatment in Denmark. More specifically, we consider an illustrative example of how one woman with a fear of falling, especially in public spaces, faced the challenges she encountered in busy urban environments through her use of *Minddistrict*, an app developed to digitally support an individual’s personal route to recovery. Minddistrict is an international company, operating within different national contexts in Northern Europe in the area of mental healthcare: in Denmark, the app is part of an internet-based therapy program on offer to people suffering from mild anxiety issues or depression. Citizens can apply online for treatment, circumventing regular health protocols based on doctor referral systems, and instead directly approach a team of professional psychologists to assess eligibility. Once accepted, treatment consists of a 12-week program, where a-synchronic textual interaction with a psychologist, online meetings, and engaging with the Minddistrict app are key components. The program functions somewhat like an online and interactive self-help book, whereby the psychologist reviews descriptions and reflections on a weekly basis, providing written support and feedback. The treatment is based on cognitive behavioural therapy and is primarily for people who suffer from mild to moderate anxiety or depression, and who have the resources to work on recovery alone. Treatment, therefore, is not designed for people with schizophrenia or other such complex psychiatric diagnoses. Throughout treatment, users independently work through a series of steps that provide them with the tools and knowledge necessary to better manage their mental health issues. The treatment is espoused as being as efficient as traditional therapy, with the added advantage of the technology being ready at hand. All you need is a smartphone, making accessibility not an issue for most. It is considered to be easy to use and treatment is relatively inexpensive, although this, of course, points to wider questions about the use of technologies to compensate for shrinking healthcare budgets and increasingly individualised healthcare practices, where the weight of responsibility for care falls to the patient (Oudshoorn 2011).

An individual’s use of the Minddistrict app was recently profiled on the website of the Danish broadcaster, DRTV (2022), and we draw on this account to think through some of our core themes on care and place outlined above. Amalie explained in a short interview how she worked with the Minddistrict app to overcome her anxieties related to falling, as they manifested in public space. The source of Amalie’s anxiety was an accident in which she fell out the back of a vehicle moving at 60 kms per hour, during an end of high school celebration. Amalie’s traumatic experience left her haunted by daily anxieties when moving through urban environments, such as shopping centres and transport stations. The simple act of walking on flights of stairs or encountering escalators in public spaces became overwhelming and distressing for her. Her DRTV interview was held at the top of a set of escalators and stairs at Odense train station, where she noted “I feel awful. I can feel how my heart rate is rising and I start to sweat. When I get closer to the stairs, my legs become very hard and [it is] very hard to move.” She continued to say that “If I get a foot wrong, I fall. When I have met these stairs here, it has been insanely frustrating. I’ve felt that I couldn't really get around as everyone else can” (DRTV 2022). The built environment, which once provided functionality and convenience, became a constant source of fear and unease. Writing down her experiences in the app as part of her treatment helped broker her anxieties, which, arguably, were neither placed within Amalie nor within the built environment, but nested within the relations between her memory of the accident and the built environment.

In this single account of how a mundane digital technology is used to connect an intense emotional experience triggered through mundane materialities and everyday urban infrastructures (a staircase, an escalator), the app serves as a powerful mediator, providing Amalie with the tools to navigate and mitigate her distressing experiences within the built environment. Agency, then, may be said to be refracted across broader assemblages, with the app affording Amalie the support to engage her surroundings in a different way. In her heightened *attentivenes*s to the environment, the app enabled Amalie to be *responsive* within her situation, thus allowing her to relate to the environment in a way that enabled care (Tronto 2015; also Duff 2011), and opened up an expanded understanding of therapeutic space (Trnka 2021). Amalie’s situation is technogeographically configured, then, with the app working to redistribute responsibilities of care in a heightened moment of distress, and to broker interdependencies between patient, professional, and place (Oudshoorn 2012). The app’s digital platform, furthermore, allows Amalie to emplace her experiences digitally, enabling her to work through her anxieties and confront her fears at her own pace, affording a different spatio-temporal aspect to the recovery process. By integrating therapy and self-reflection into her daily life, the Minddistrict app empowers Amalie to actively address her anxieties. When asked by a journalist whether she missed having any physical interaction during her treatment, she promptly replies “no, I haven’t at any point needed it. I think I have received the support I needed online. I have not been dependent on having to show up at a physical place. I have been able to do it at my own pace” (DRTV 2022).

Thinking beyond this example of Amalie’s experience in a Danish context, there are striking similarities between the Minddistrict app and how other online apps have been considered to affect the relational qualities of place and care elsewhere. In particular, Trnka’s understanding of the multi-sited nature, both online and material, of therapeutic assemblages draws from conversations with medical professionals in New Zealand, such as a clinical psychologist who discussed her experience of developing an anti-anxiety app (2021). This app was developed in response to some of the structural problems around accessing psychological services in New Zealand, due to unprecedented high levels of demand: memorably describing her app as “a pocket psychologist … providing support when there is none,” the psychologist who spoke with Trnka designed her app to offer the type of coping exercises hitherto taught in clinical encounters, in institutional spaces (2021). These are the types of exercises noted by Amalie in her account of addressing anxieties in Odense, and our Danish example offers a similarly notable example of how digital technologies hold the capacity to transform any place—not just homes, but public places of leisure and transport, the typical non-places of cities that we are meant to pass through quickly rather than dwell within—into sites of care (Ivanova 2020a). By the quick action of recording her emotions within the Minddistrict app, Amalie was able to recover through a high-point of anxiety without even the need for synchronous clinical contact, at a distance or in-person, with a psychologist. The specificity of place, and liveliness of digital technology within the everyday (Leszczynski 2019), coalesced at that moment in time to enact a “technogeographical configuration of care” (Oudshoorn 2012: 129), albeit one that looks very different to traditional models of care that are premised on co-present and collaborative practices between patients and professionals (Mol 2008).

The account of the Minddistrict app, premised as it is on a back-stage team of psychologists responding at a distance (even if not necessarily simultaneously) with individuals who can self-manage that moment of emotional distress, does illustrate “that the care a person exercises – for him- or herself or for others – is never independent of the kinds of infrastructures that contribute to this care” (Langstrup et al. 2013, 53). Thinking through Amalie’s account, we see the very material infrastructures of place (the stairs that trigger the distress) as implicated with, and acting alongside, the digital infrastructures that ostensibly re-configure our sense of place (the app, accessed through smartphone technologies). However, we need to consider not just the kinds of infrastructures that can be located in particular places, but rather to understand *places* as infrastructures. Drawing on understandings of infrastructure as the very bases upon which social practices are enabled, Ivanova and colleagues argue that we should think of places as inherently infrastructural in character, “working by proxy – doing work and igniting action elsewhere” (2019, 145). In the Odense train station, we see that Amalie’s ability to engage her therapeutic needs in place allows her to constantly re-work her sense of agency, opening up the question of where care takes place. This example shows how the everyday, quotidian use of mundane spaces is central to understanding how multi-sited assemblages of care are continuously constituted and reconstituted—not least within wider logics of care that are shifting too, towards more individualised patient cultures (Mol 2008), where an increasing weight of responsibility for self-monitoring illness and anxiety is facilitated through digital technologies (Oudshoorn 2011).

Ivanova et al.’s analysis of place-by-proxy and, indeed, Amalie’s account challenge us to think hard about the porosity of boundaries of place, care, and their intersections. Langstrup et al. are surely right to consider care encounters as corporate in nature, even when their appearance is of an individual process of self-management (2013), as Amalie’s account implies. The clinical psychologist who spoke to Trnka about her anti-anxiety app indicated that it was designed to substitute for face-to-face encounters when needed, but in ways that “indexed back to the clinic,” engaging its users with professional services and, thus, re-validating their importance (2021). In our reading of Amalie’s account, we might consider the potency of handheld digital technologies in bringing the same clinical imaginaries that we earlier suggested as haunting the experience of telecare in domestic spaces (Weiner and Will 2018). Moreover, we can—and should—be critical of the wider structural issues that the development of apps such as Minddistrict are implicated in, with such technologies working within the context of an erosion of logics of care that are premised on collaboration between patient and practitioner rather than individualising rhetorics of patient choice (Mol 2008). Within such contexts, this is care on a budget, where the user of the app is configured within ubiquitous technological networks and treated within narrowly defined measures of mental health (Banner 2022).

And yet, we return to Amalie, and her quiet confirmation that she had received the support she needed online—“I have not been dependent on having to show up at a physical place. I have been able to do it at my own pace” (DRTV 2022). These individual cases may point to a dissonance between the intention of developers who design such anti-anxiety apps to re-inscribe the authority of the clinic, and the use of the app by users who see this differently, in terms of their agency to interact with care within everyday encounters and environments. What Amalie’s account does signal is the radical expansiveness and contingency of space (Massey 2005), which leads us to a less functional and less bounded understanding of place, with care encountered, enabled, and experienced in spaces that are alive with technologies of the ordinary rather than haunted by previous imaginaries (Leszczynski 2020). As Oudshoorn’s research suggests (2011), some places facilitate the incorporation of clinical technologies, whereas other places resist them, and we should remember that this process is neither pre-determined, nor settled. Our tracing of the movement of care mediated through digital technologies—out of the clinic, beyond the home, and into the ordinary spaces of the city—suggests that we must pay attention to the clinical logics that may accompany this process, and how these are experienced, accommodated, or avoided. In other words, our understanding of the *where of care* is always and already related to questions of who are involved, when, and how their relations are composed.

## Conclusion

In this paper, we have sought to extend debates on the intersections between place and care, with a focus on the use of everyday digital technologies in ordinary urban environments, in order to move debates on telecare on from their previous, and understandable, locations within domestic settings alone (Langstrup 2013; Wiener and Will 2018). Specifically, we introduced an account of how the Minddistrict app was used in a busy urban place to negotiate feelings of anxiety (that were triggered through the built environment), in order to explore the dispersal of care-infrastructures beyond clinical environments and into public spaces (Langstrup 2013), facilitated by the use of mundane smartphone technologies. We considered the role of these technologies in brokering new technogeographical configurations of care (Oudshoorn 2011), and remaking the places in which they are used in an infrastructural sense, in that apps such as Minddistrict work to integrate the public spaces of the city within wider care networks, making them *places-by-proxy* that act as conduits to sources of support, within a wider milieu of care (Ivanova et al. 2019).

An aim of this paper has been to open up a sense of care in contemporary lives and cities as portable, facilitated by mobile digital technologies that, through the ubiquity of their use, transform previous understandings of the therapeutic that were tied to particular single sites (the clinic, the home). Instead, researchers must be alert to a more diverse range of places in which care is configured—the therapeutic is, indeed, a multi-sited assemblage (Trnka 2021). As Trnka explains (2021), conceptualising multi-sited spaces of care means that these “assemblages are characterised by fluidity and inter-relationality, with multiple sites constituted in relation to one another”, and with experiences of support felt in particular places only understood in relation to their situatedness within other places in a wider care network. This can, of course, lead to a diffusion of certain logics between different types of spaces—as we have argued, the existing literature shows us how clinical imaginaries haunt domestic spaces once they become recruited by telecare technologies as places for managing chronic illness (Weiner and Will 2018). But, as we have also argued, it is not necessarily the case that digital technologies act simply as extensions of the clinic, re-inscribing its authority, even when this has been an intention behind their design (Trnka 2021). Doctors’ surgeries and psychiatrists’ clinics may still be important within the experience of anxiety and mental health more generally, but only as individual infrastructural elements within the wider milieu of care. As Puig de la Bellacasa has argued, a focus on relations between humans and non-humans leads to a displaced sense of care (2017); moreover, in our example, we can see not only a dispersal of care through different spaces, but also a decentring of clinical expertise, and a more nuanced picture of how agency is enacted. We would therefore argue that technologies should not be considered as tools for adjusting the behaviour of people within places to medicalised norms, but rather in an infrastructural way, allowing people to engage with their surroundings in active ways and emplacing their experiences digitally. Indeed, navigating and brokering the broader ecologies of distress in the city through the use of digital technologies, which may, in turn, evoke and enact a wider milieu of care, shows how we might begin to consider many contemporary place-care relations as being alive within the ordinary materialities of the everyday. As Tronto has argued, we are already and always immersed in relations and networks of care (2018).

We hope this paper’s analysis of one woman’s account of using an anti-anxiety app in public space demonstrates the value of moving the lens of enquiry from the perspective of the individual to a wider constellation of human and non-human actors, technologies, and places (Langstrup et al. 2013). Of course, this focus should not preclude a wider unease about the ways in which digital technologies can be used to compensate for falling healthcare budgets, and to supplant logics of care that place healthcare practitioners and patients within co-present cultures of collaboration (Mol 2008; also Frank 2004). *Minddistrict* is only one example of many apps, such as *Betterhelp* and *Talkspace*, which we should be concerned about because of the ease with which they extend psychiatric power into digital space, with individual patients enrolled within big 

data ecosystems notable for their consumer and racial logics (Banner 2022). And although contemporary digital solutions promise smooth, resource-efficient, and accessible treatment, both in policy development and practice innovation, under the surface of such rhetoric we must consider the “more-than-digital” implications these solutions bring with them. That is, these digital solutions are, at root, premised on the need for informal support from family and friends, tech-literacy on the part of its users, and, indeed, the reconfiguration of social relations and redistribution of responsibilities in often highly gendered ways (Oudshoorn 2011). However, these are arguments to take further in future research; for this paper, we have been especially concerned with tracing imaginaries and practices of care that are “pushing the notion of place into new conceptual grounds” (Ivanova 2020b, 1306), where familiar place/care ontologies are disrupted through the influence of technologies within places where care is sought and experienced. Our study has worked with an understanding of place as “an infrastructural achievement” (Ivanova et al. 2019, 156), agentic in its prompting of action elsewhere, at-a-distance, and asynchronously. Put simply, mundane technologies change how we conceive of care, and place reconfigures how we care (Oudshoorn 2011). Separately, but especially together, the entanglements of digital technologies and ordinary places prompt us to entirely reconsider the *where* of care.

## Data Availability

Not applicable.
